# Coordinated Application of Nitrogen and Sulfur Synergistically Enhances Grain Yield and Grain Protein Concentration of Rice by Regulating Plant Growth

**DOI:** 10.3390/plants15071058

**Published:** 2026-03-30

**Authors:** Honglin Wang, Jianan Fu, Huadong Gong, Linyan Kuang, Yuzhe Song, Zhaoyue Ma, Liqiang He, Bohan Xu, Shuai Cui, Shuoran Liu, Zhongqing Zhang, Qiang Gao

**Affiliations:** 1 School of Tropical Agriculture and Forestry, Hainan University, Haikou 570228, China; 2 College of Resource and Environmental Sciences, Jilin Agricultural University, Changchun 130118, China

**Keywords:** nutrient management, plant growth regulation, nitrogen, sulfur, rice

## Abstract

Simultaneous achievement of high yield and excellent quality in rice is essential for food security and human health. The coordinated application of nitrogen (N) and sulfur (S) can effectively increase the grain yield (GY) and grain protein concentration (GPC) of rice. A two-season field experiment was conducted to investigate the synergistic effects of combined N and S application on the GY and GPC of rice. This study employed four N rates (0, 120, 180, and 240 kg ha^−1^, designated as N0, N1, N2, and N3, respectively) and four S rates (0, 30, 45, and 60 kg ha^−1^, designated as S0, S1, S2, and S3, respectively) using two rice cultivars: Jiujiuxiang (JJX) and Jiuxiangyou (JXY). The experimental results demonstrate that N and S exert significant effects on the GY and GPC of rice, with notable interactive effects between these two nutrient elements. The synergistic fertilization of N and S enhanced the GY by improving rice plant photosynthesis and dry matter accumulation while increasing GPC through elevated cysteine concentration in grains. Compared to the unfertilized treatment, the GY of the JJX cultivar showed increases of 68.3–143.2% (Season I) and 59.4–133.4% (Season II) under combined N and S applications, while the GY of the JXY cultivar increased by 53.2–144.1% (Season I) and 66.0–192.9% (Season II). Similarly, the GPC of the JJX cultivar showed increases of 7.5–43.4% (Season I) and 5.7–43.9% (Season II) under combined N and S applications, while the GPC of the JXY cultivar increased by 13.1–66.7% (Season I) and 13.3–61.0% (Season II). Overall, whether on the JJX or JXY cultivars, the application of 180 kg ha^−1^ of N combined with 45 kg ha^−1^ of S (i.e., the N2S2 treatment) synergistically enhances GY and GPC in rice. The synergistic fertilization of N and S synergistically enhances both rice yield and nutritional quality by regulating plant growth dynamics, which meet the requirements for healthy and sustainable development in rice production systems.

## 1. Introduction

Rice (*Oryza sativa* L.), as the predominant staple crop for more than half of the world’s population, is an important source of dietary energy and protein; its yield and nutritional quality are directly related to global food security and human health [1,2]. Amidst population growth and evolving nutritional consumption patterns, achieving the synergistic enhancement of both rice yield and nutritional quality has emerged as a pivotal objective for the sustainable development of modern agriculture. As the major components in grains, starch and storage protein are primary determinants of the yield and nutritional quality of rice [3]. Starch, the predominant storage in grain (~80%), supplies the main energy source for humans and is consequently the leading determinant of grain yield (GY) [4]. Storage proteins are the second largest nutrient components of rice endosperm, accounting for approximately 8–10% of the dry weight of grain [5]. By contrast, storage protein has little effect on GY due to its low content in grains. However, storage protein provides different types of amino acids, thereby influencing the nutritional quality of rice [6]. Therefore, enhancing the photosynthetic efficiency of leaves and promoting starch accumulation represents the optimal strategy for achieving high GY, while increasing grain protein concentration (GPC) is crucial for increasing the nutritional quality of rice. However, in conventional high-yield cultivation systems, GY is often negatively correlated with GPC of rice, meaning that high GY is typically associated with a relative reduction in GPC [7]. Analysis of multiple genotypes revealed that a 1.0 t ha^−1^ increase in GY was associated with an average reduction of 0.46 percentage points in GPC of rice [8]. This “dilution effect” restricts the simultaneous improvement of GY and nutritional quality in rice. Thus, how to break this trade-off relationship through scientific nutrient management strategies is an important challenge in current rice cultivation research.

Nitrogen (N) is one of the principal macronutrients essential for crop growth, development, and reproduction, accounting for approximately 2–4% of the plant’s dry weight [9,10]. As an essential component of various bioactive molecules, including proteins, amino acids, chlorophyll, nucleic acids, adenosine triphosphate, and vitamins, N plays a critical role in numerous biological processes of plants and determines crop yield and nutritional quality [11]. N is directly involved in photosynthesis, biomass accumulation, tillering activity, spikelet formation, grain development, and grain protein synthesis [12]. Previous studies have found that N application enhanced the photosynthetic capacity, dry matter accumulation, and GY in rice [13,14]. The yield components can directly reflect the GY of rice, with N fertilization exerting differential effects on the various yield constituents. Previous research has demonstrated that increasing N application enhances the number of effective panicles, spikelets per panicle, and seed-setting rate, while the 1000-grain weight exhibits a slight decline with elevated N application [15,16]. Generally, the GPC is an important factor in evaluating the nutritional value of rice, and N fertilizer management has a profound impact on the GPC. The N translocation within the plant regulates its allocation between the vegetative organs (such as leaves and stems) and the reproductive organs (such as panicles and grains), which is particularly important for efficient grain filling and N accumulation in grains [17,18]. During the grain filling stage, adequate N supply promotes the N redistribution from source organs to sink tissues and effectively enhances the GPC, thereby improving the nutritional quality of rice [19]. Multiple studies have exhibited that there was a parabolic relationship between GY and N rates, which means the excessive N application may result in rice yield stagnation and reduction [20,21,22]. Furthermore, excessive N fertilization can lead to plant lodging [23], diseases and pests [24], N loss [25], and a decline in grain quality of rice [26]. However, the persistent pursuit of higher yields and returns by farmers has led to excessive N application, which continuously threatens the simultaneous improvement of GY and nutritional quality in rice [27,28]. Therefore, it is imperative to formulate appropriate N management strategies for synergistic enhancement of yield and nutritional quality in rice.

Sulfur (S) represents the fourth major plant nutrient element following nitrogen, phosphorus, and potassium [29], with its concentration in plant tissues ranging from 0.1% to 1.5% of dry weight, depending on growth stage and species [30]. As an essential component of functional substances in plants (such as coenzymes, prosthetic groups, amino acids, and proteins) [31], S not only plays a critical role in crop yield but also regulates nutritional quality through the synthesis of S-containing amino acids, including cysteine and methionine [32,33]. Numerous studies demonstrate that adequate S supply enhances crop yield and nutritional quality [34,35,36,37], while playing a critical regulatory role in plant responses to abiotic stress [38,39]. However, in recent years, due to the combined effects of reduced atmospheric S deposition, application of high-purity low-S synthetic fertilizers, low S returns in livestock manure, decreased usage of S-based pesticides, cultivation of crop varieties with high yield potential and high nutrient demands, and agricultural intensification, these factors have collectively triggered a global S deficiency in soils [40,41,42]. Previous research indicated that in the absence of adequate S supply, crops cannot fully realize their potential in terms of yield and protein accumulation, nor can they efficiently utilize applied N, which is attributed to the indispensable S requirement for protein and enzyme synthesis and the regulation of physiological processes [43,44]. Field experiment results demonstrate that under S-deficient conditions, rice GY decreased by 35% [45], while GPC was reduced by 15.4% [46]. The S metabolism and requirement of plants are closely associated with N nutrition, while N metabolism is also strongly influenced by the S status of plants [47]. Similarly to the tight coupling between carbon assimilation pathways and nitrate assimilation in plants, the assimilation pathways of S and N are also intricately interconnected, with the availability of one element modulating the utilization of the other [48]. In field experiments, S application increased GY and GPC by 7% and 22%, respectively, and the enhanced GY and GPC response to S fertilization was associated with a higher uptake of N before anthesis [49].

In conclusion, both N and S play pivotal regulatory roles in rice GY and GPC, with a significant interactive effect observed between them. Furthermore, the GY and GPC of different rice cultivars exhibit varied responses to N and S fertilization [26,50]. Therefore, a field experiment with different N and S application rates on two rice varieties was established to investigate the regulating effect of N and S in synergistically enhancing the GY and GPC of rice. In this study, rice GY and its components, leaf net photosynthetic rates, plant dry matter, GPC and cysteine concentrations in grains were measured and analyzed to explore the influences of coordinated fertilization of N and S on the yield and nutritional quality of rice, with the aim of providing valuable references for the regulation of rice growth and development and scientific fertilization.

## 2. Results

### 2.1. Grain Yield and Its Components

**Figure 1:**
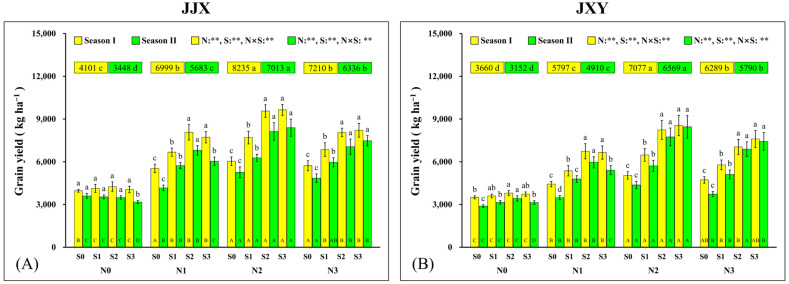
Grain yield of two rice varieties under different N and S application rates. (**A**) Grain yield of the JJX variety; (**B**) grain yield of the JXY variety. The lowercase letters above the bars denote statistically significant differences between different S application rates at the same N level, and different uppercase letters in the bars denote statistically significant differences between different N application rates at the same S level. The number within the top boxes of each set of bar charts indicates the average value of the grain yield at different N application levels, and the lowercase letters after the number indicate that the grain yield has significant differences at different N application levels. The variance analysis used two-way ANOVA, and ** denotes significance at *p* < 0.01.

As shown in Figure 1, the trends in rice GY changes in the two planting seasons are consistent, and the GY responses of the two rice varieties (JJX and JXY) exhibited a similar regularity. The results showed that both N and S had significant effects on GY of rice, with a significant interaction between the two elements. Compared with the N0 level, N1, N2, and N3 significantly increased the GY of rice, but N3 was significantly lower than N2, indicating that excessive N application inhibited the GY. In the second planting season, the effect of soil N consumption and S accumulation became prominent. The test results of soil samples after the first planting season’s harvest showed that, under no N application (N0), the soil’s available N content decreased by 22.6% compared with that before planting (84.93 mg kg^−1^), with a value of 65.71 mg kg^−1^. Concurrently, with the increase in S application rate, the available S content in the soil samples increased by 83.1%, 124.4%, and 170.8% relative to the initial values (10.58 mg kg^−1^), corresponding to measured values of 19.37 mg kg^−1^, 23.74 mg kg^−1^, and 28.65 mg kg^−1^, respectively. At the N0 level, the GY of JJX continuously decreased with an increase in S application (compared with S0, the GY of S3 decreased by 11.8%), while that of JXY first increased (compared with S0, the GY of S2 increased by 18.8%) and then decreased (compared with S2, the GY of S3 decreased by 8.3%) with an increase in S application, indicating that the two rice varieties have different sensitivities to S applications under the no-N application. At the N1 level, the GY of the two rice varieties increased first (compared with S0, the GY of S2 increased by 63.3% and 71.4%) and then decreased (compared with S2, the GY of S3 decreased by 11.2% and 9.3%) with an increase in S application, indicating that excessive S application under insufficient N application inhibited the GY. At the N2 and N3 levels, the GY of the two rice varieties increased with an increase in S application, but S3 (the GY of JJX and JXY was 8391.50 kg ha^−1^ and 8450.39 kg ha^−1^, respectively) was not significantly higher than S2 (the GY of JJX and JXY was 8126.64 kg ha^−1^ and 7741.92 kg ha^−1^, respectively), indicating that excessive S application no longer increased the GY under sufficient N application conditions. The foregoing results demonstrate that both supra-optimal and suboptimal N and S application rates significantly reduced the GY of rice. Accordingly, N2S2 constitutes the optimal N and S nutrient management regimen for enhancing GY in rice.

The results of the yield component analysis indicated that the effective panicles, grain numbers per panicle, and grain filling percentage of the two rice varieties were significantly affected by the planting season, except for the 1000-grain weight (Table 1). N and S have significant effects on all yield components, and there is a significant interaction between the two elements on 1000-grain weight and grain filling percentage (except for JXY). There were no two-factor and three-factor interactions among planting seasons, N, and S for all yield components. The effective panicles and grain numbers increased with rising N application rates, whereas the 1000-grain weight and grain filling percentage decreased. The balance among yield components plays a critical role in determining the GY response to N fertilization. At high N application levels (N3), the reduction in grain weight resulting from delayed grain filling constitutes a significant contributing factor to the decline in GY of rice. Under conditions without N application, moderate S application can increase the grain numbers, but excessive S application will reduce the grain numbers, and the 1000-grain weight will continuously decrease with an increase in S application. S application had a positive impact on all yield components at N application, which was an important manifestation of the increase in the GY of rice. Notably, there was no statistically significant three-way interaction among planting season, N, and S on yield components. However, during the second planting season, insufficient N application coincided with a reduction in 1000-grain weight under high S application (S3), despite the absence of statistically significant differences relative to the S2 level. Nevertheless, given the substantial number of grains per unit area, this marginal decline in 1000-grain weight likely contributed to a meaningful reduction in overall GY of rice. When N application was sufficient, further S application coordinatively enhanced the effective panicles, grain numbers per panicle, 1000-grain weight, and grain filling percentage, which aligns with the observed increase in final GY of rice.

### 2.2. Leaf Photosynthesis

**Figure 2:**
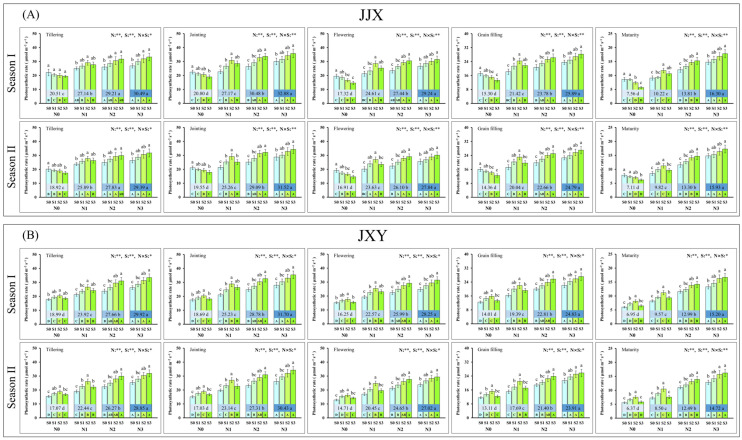
Leaf photosynthetic rate of two rice varieties under different N and S application rates. (**A**) Leaf photosynthetic rate of the JJX variety at different plant growth stages; (**B**) leaf photosynthetic rate of the JXY variety at different plant growth stages. The lowercase letters above the bars denote statistically significant differences between different S application rates at the same N level, and the different uppercase letters in the bars denote statistically significant differences between different N application rates at the same S level. The number within the central boxes of each set of bar charts denotes the average value of the photosynthetic rate at different N application levels, and the lowercase letters after the number indicate that the photosynthetic rate has statistically significant differences at the different N levels. Variance analysis used two-way ANOVA, ** denotes significance at *p* < 0.01, and * denotes significance at *p* < 0.05.

Leaf photosynthesis was the primary physiological basis for crop yield formation; therefore, we conducted dynamic measurements of the net photosynthetic rate in rice leaves across key growth stages in two planting seasons. The changing trend of the net photosynthetic rate of rice leaves across key growth stages was highly consistent in two planting seasons, and the responses among different varieties are also similar (Figure 2). The dynamic monitoring results demonstrated that N and S application significantly affected the leaf net photosynthetic rate of rice, with a significant interaction between the two nutrients. At each key growth stage of rice development, the leaf net photosynthetic rate exhibits a continuous increase in response to escalating N application levels. Under the experiment without N application (N0), the net photosynthetic rate of the leaves in the two cultivars exhibited differential responses with increasing S application, indicating differential sensitivity to S between the cultivars under N deficiency. In the absence of N application (N0), the leaves’ net photosynthetic rate of the JJX cultivar exhibited a continuous decline with increasing S application rates, whereas that of the JXY cultivar initially increased (S2) before subsequently decreasing (S3). At lower N application levels (N1), the leaf net photosynthetic rate of both cultivars initially increased (S2) and then declined (S3) with rising S application rates. This finding indicated that under limited N availability, appropriate S fertilization can enhance leaf photosynthesis, whereas excessive S application exerts a detrimental effect. Under sufficient N application (N2 and N3), the leaf net photosynthetic rate in both cultivars continuously increased with elevated S application rates, but there was no statistically significant difference between S3 and S2 levels, indicating that moderate S fertilization further enhanced leaf photosynthesis when N was abundant. Leaf net photosynthetic rate and GY of rice exhibited consistent responses to N and S application, which explains the reason for the increased GY from the perspective of plant physiology.

### 2.3. Plant Dry Matter

**Figure 3:**
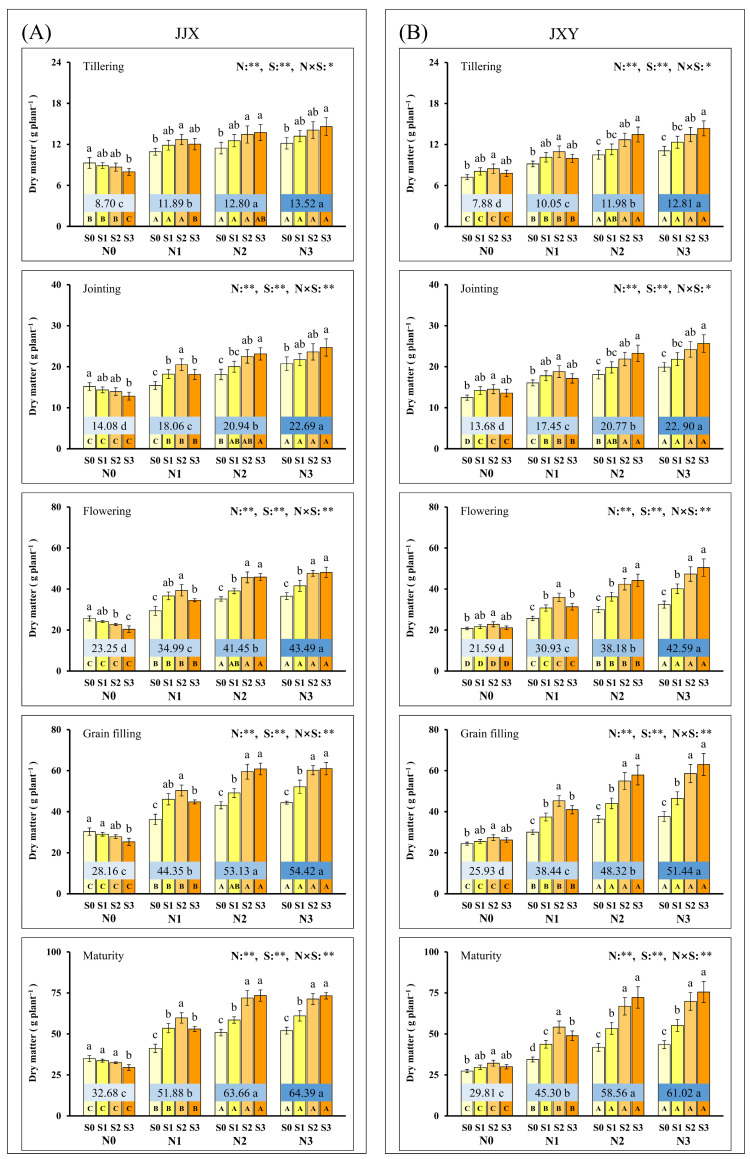
Dry matter of two rice varieties under different N and S application rates. (**A**) Dry matter of the JJX variety at different plant growth stages; (**B**) dry matter of the JXY variety at different plant growth stages. The lowercase letters above the bars denote statistically significant differences between different S fertilization rates at the same N level, and the different uppercase letters in the bars denote statistically significant differences between different N fertilization rates at the same S level. The number within the central boxes of each set of bar charts denotes the average value of the dry matter at different N fertilization levels, and the lowercase letters after the number denote that the dry matter has statistically significant differences at different N levels. Variance analysis used two-way ANOVA, ** denotes significance at *p* < 0.01, and * denotes significance at *p* < 0.05.

The accumulation of plant dry matter directly reflects the storage of photosynthetic assimilates and exhibits a close correlation with the GY of rice. Accordingly, we conducted dynamic monitoring of dry matter accumulation in plants during the second planting season. The experimental results indicated that N and S applications exerted significant effects on plant dry matter accumulation across all key growth stages, with a significant interactive effect observed between these two factors (Figure 3). The response of dry matter accumulation in plants to N and S application was highly similar to that of the leaf net photosynthetic rate. The dry matter accumulation in plants exhibited a continuous increase with rising N application rates, with both cultivars demonstrating a consistent response trend. The response of plant dry matter accumulation to S application rates was inconsistent across different N supply levels. In the absence of N application (N0), the dry matter accumulation in the JJX cultivar progressively decreased with increasing S application rates, and the minimum values recorded across five key growth stages under the S3 level were 7.98, 12.84, 20.44, 25.37, and 29.54 g plant^−1^. The plant dry matter accumulation of the JXY variety initially increased (S2) and then decreased (S3) with S fertilization rates, and all treatments reached the maximum values at the S2 level, which were 8.46, 14.52, 22.81, 27.43, and 32.19 g plant^−1^. At lower N application rates (N1), a moderate S supply (S2) promoted the accumulation of plant dry matter, whereas excessive S application (S3) inhibited dry matter accumulation. Under moderate (N2) and excessive (N3) N application, S application can further increase the dry matter accumulation of plants, indicating that the synergistic effect of N and S was fully exerted. Adequate accumulation and efficient translocation of dry matter in plants constitute a key physiological determinant of increased GY of rice. It is noteworthy that during the rice maturity stage, there was no statistically significant difference in the accumulation of dry matter between the N2 and N3 levels, whereas the GY under the N3 level was significantly lower than that of the N2 level. This finding indicates that N application at the N3 level leads to a greater accumulation of dry matter predominantly in the stalks (including leaves), without an effective translocation to the grains of rice. Considering both the dynamic regulatory roles of N and S in dry matter accumulation and the economic efficiency of fertilizer application, the N2S2 combination represents the optimal integrated nutrient management strategy for N and S.

### 2.4. N and S Concentrations in Leaves at Different Plant Growth Stages

**Figure 4:**
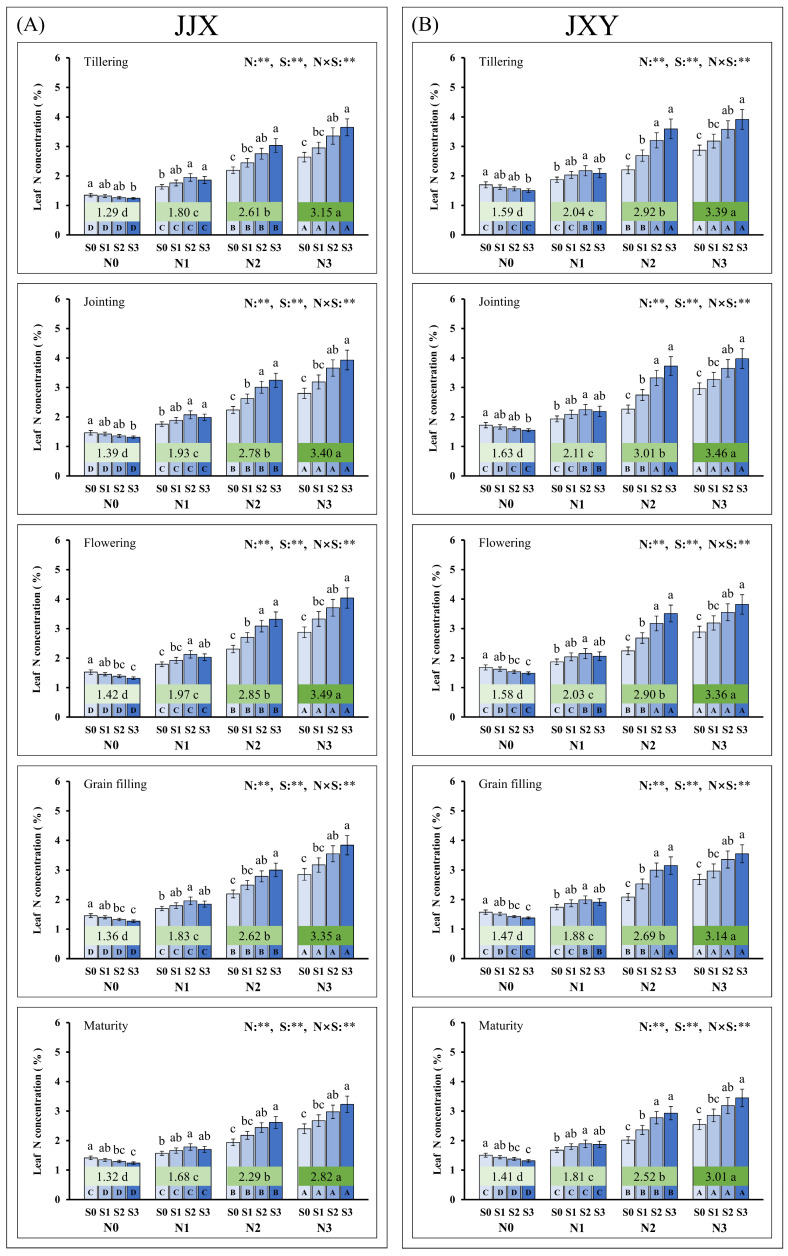
Leaf N concentration of two rice varieties under different N and S fertilization rates. (**A**) Leaf N concentration of the JJX variety at different plant growth stages. (**B**) Leaf N concentration of the JXY variety at different plant growth stages. The lowercase letters above the bars denote statistically significant differences between different S fertilization rates at the same N level, and different uppercase letters in the bars denote statistically significant differences between different N fertilization rates at the same S level. The number within the central boxes of each set of bar charts denotes the average value of the N concentration at different N fertilization levels, and lowercase letters after the number denote that the N concentration has statistically significant differences at different N fertilization levels. Variance analysis used two-way ANOVA, and ** denotes significance at *p* < 0.01.

The absorption of N and S by plants has a significant impact on photosynthesis, dry matter accumulation, and GY of rice. To further investigate the effects of N and S on the plant growth of rice, we monitored the N and S concentrations in the leaves of rice plants during key growth stages in the second planting season (Figure 4). The experimental results demonstrated that both N and S exert significant effects on leaf N concentration in rice, and a significant interaction between these two nutrients was observed. The effect of N and S on leaf N concentrations exhibited no significant genotypic variation, indicating that this influence is universally applicable. The experimental results indicated that the leaf N concentration of rice consistently demonstrates a progressive increase with elevated N application rates across each key growth stage. Under conditions without N application (N0), the N concentration in rice leaves decreased progressively with increasing S application rates, and the minimum values all occurred at the S3 level (the values of JJX were 1.24%, 1.31%, 1.32%, 1.27%, and 1.24%; the values of JXY were 1.50%, 1.55%, 1.48%, 1.38%, and 1.31%), indicating that S application inhibits N uptake in plants with N deficiency. When N application was insufficient (N1), moderate S application (S2) increased the N concentration in leaves (the maximum values of JJX were 1.95%, 2.07%, 2.13%, 1.96%, and 1.78%; the maximum values of JXY were 2.18%, 2.25%, 2.15%, 1.99%, and 1.89%), while excessive S application (S3) still reduced the absorption of N by plants. Although no significant difference was observed between the S3 and S2 treatments, excessive S application is not recommended from a biological and economic perspective. Under sufficient N application conditions (N2 and N3), leaf N concentrations in rice increased continuously with increasing S application rates, but there was no significant difference between S3 and S2 levels, suggesting that moderate S supply further promoted N uptake when N availability is adequate. The changing trend of N concentration in leaves is highly consistent with the net photosynthetic rate, confirming the regulatory role of N in photosynthesis and the dry matter accumulation of plants.

**Figure 5:**
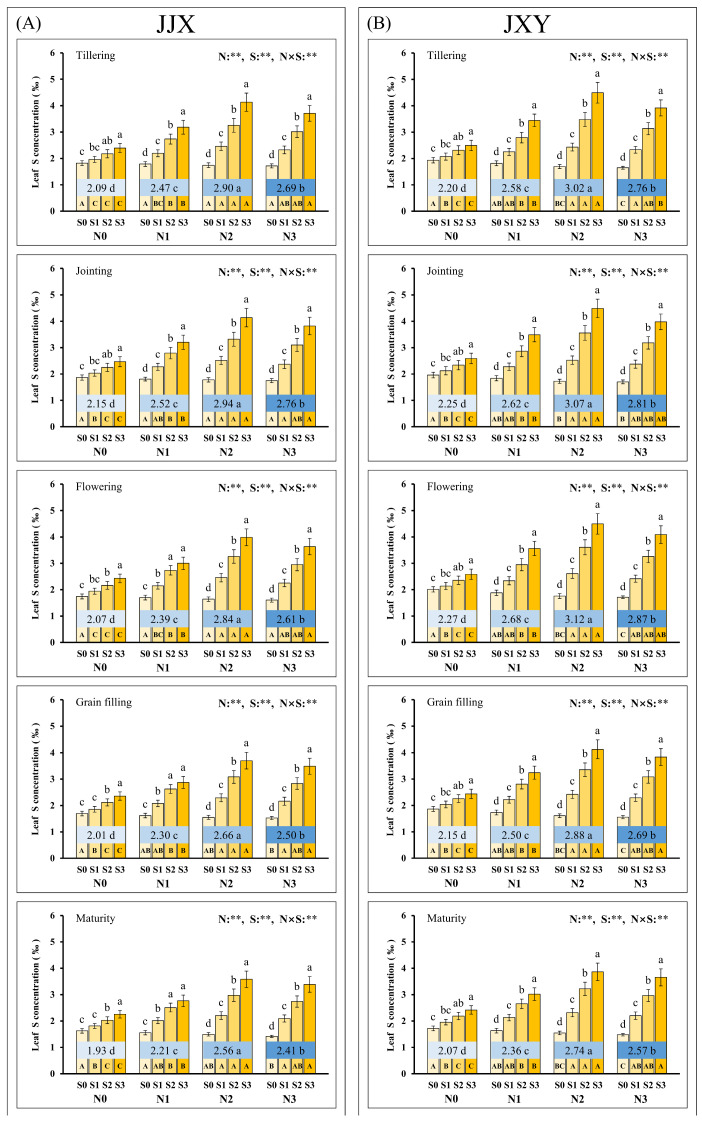
Leaf S concentration of two rice varieties under different N and S fertilization rates. (**A**) Leaf S concentration of the JJX variety at different plant growth stages. (**B**) Leaf S concentration of the JXY variety at different plant growth stages. The lowercase letters above the bars denote statistically significant differences between different S fertilization rates at the same N level, and the different uppercase letters in the bars denote statistically significant differences between different N fertilization rates at the same S level. The number within the central boxes of each set of bar charts denotes the S concentration at different N fertilization levels, and the lowercase letters after the number denote that the S concentration has statistically significant differences at different N fertilization levels. Variance analysis used two-way ANOVA, and ** denotes significance at *p* < 0.01.

The dynamic monitoring results of two varieties revealed that N and S application significantly influenced the leaf S concentration of rice, with a statistically significant interaction between the two nutrients (Figure 5). The S concentration in leaves showed a first increasing (the maximum average values all occurred at the N2 level, and the values of JJX were 2.90‰, 2.94‰, 2.84‰, 2.66‰ and 2.56‰; the values of JXY were 3.02‰, 3.07‰, 3.12‰, 2.88‰ and 2.74‰) and then significantly decreasing (N3) trend with rising N application rates, indicating that appropriate N application is conducive to S accumulation, while excessive N application inhibits S absorption by plants. Under all N application levels, leaf S concentrations continuously increased with rising S application rates. However, in the absence of N fertilization, the incremental increase in leaf S concentration due to S application was relatively limited, indicating that the S uptake potential in plants was not fully exploited under N-deficient conditions. Consequently, appropriate N input can further promote S uptake in plants.

Based on the above results, a comprehensive analysis of GY, leaf photosynthesis, accumulation of plant dry matter, and leaf N and S concentrations indicates that the N2S2 combination is the optimal fertilization amount to ensure high GY in rice.

### 2.5. Grain Protein Concentration (GPC)

**Figure 6:**
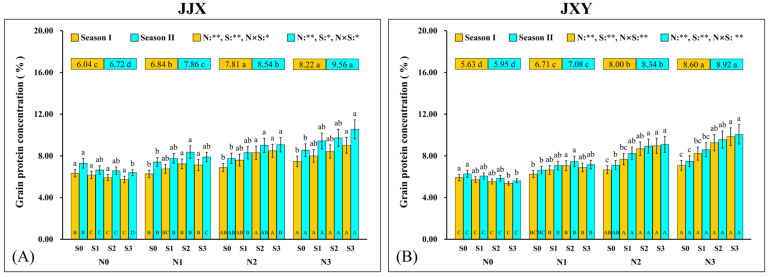
Grain protein concentration of two rice varieties under different N and S application rates. (**A**) Grain protein concentration of the JJX variety. (**B**) Grain protein concentration of the JXY variety. The lowercase letters above the bars denote statistically significant differences between different S fertilization rates at the same N level, and the different uppercase letters in the bars denote statistically significant differences between different N fertilization rates at the same S level. The number within the top boxes of each set of bar charts denotes the average value of the grain protein concentration at different N fertilization levels, and the lowercase letters after the number denote that the grain protein concentration has statistically significant differences at different N fertilization levels. Variance analysis used two-way ANOVA, ** denotes significance at *p* < 0.01, and * denotes significance at *p* < 0.05.

The GPC of rice is an important manifestation of N accumulation and nutritional quality. The experimental results from two consecutive planting seasons demonstrated that both N and S exert significant effects on GPC, with a notable interaction observed between these two factors (Figure 6). The GPC changing trends in the two varieties were highly consistent, suggesting no genotypic variation in the regulatory effects of N and S on GPC. Specifically, the GPC of both varieties consistently increased with N application rates across the two planting seasons, reaching maximum values at the N3 level. Compared with the N0 treatment, the GPC of JJX across the two planting seasons under the N3 level increased by 36.1% and 42.3%, and that of JXY increased by 52.8% and 49.9%. In the absence of N fertilization (N0), GPC exhibited a continuous decline with increasing S application rates across both planting seasons, indicating that S fertilization inhibits N uptake of grains under N-deficient conditions. Under the absence of N treatment (N0), compared to the S0 level, the GPC of the S3 level in the JJX cultivar decreased by 9.4% and 12.3% across the two planting seasons, and that of the JXY cultivar declined by 9.3% and 10.7%. Under lower N application treatment (N1), the GPC of both cultivars exhibited an initial significant increase (the maximum values in two planting seasons all occurred at the S2 level, the values of JJX were 7.23% and 8.36%, and the values of JXY were 7.06% and 7.47%) followed by a slight decline (S3) with S application rates, but the values exhibited no significant difference between the S3 and S2 levels. Under both optimal N application (N2) and excessive N application (N3) conditions, the GPC of both cultivars exhibited a continuous increase with increasing S application rates, but there was no significant difference between the S3 and S2 levels. This result indicates that S application beyond the optimal level does not further enhance GPC. It is important to emphasize that the GPC at the N3 level (the values of JJX were 8.22% and 9.56% in two planting seasons, and the values of JXY were 8.60% and 8.92%) exceeds that of the N2 level (the values of JJX were 7.81% and 8.54% in two planting seasons, and the values of JXY were 8.00% and 8.34%), but the GY at the N3 level is significantly lower than that at the N2 level. This led to a final grain protein yield (GY × GPC) at the N3 level (the values of JJX were 592.7 kg ha^−1^ and 605.7 kg ha^−1^ in two planting seasons, and the values of JXY were 540.9 kg ha^−1^ and 516.5 kg ha^−1^) that is lower than that at the N2 level (the values of JJX were 643.2 kg ha^−1^ and 598.9 kg ha^−1^ in two planting seasons, and the values of JXY were 566.2 kg ha^−1^ and 547.9 kg ha^−1^). Therefore, the N2 level represents the optimal N application rate for synergistically enhancing GY and GPC. Considering the effects of combined N and S on GPC and the economic efficiency of fertilization, the N2S2 combination is a recommended N and S nutrient management measure.

### 2.6. Grain S Concentration

**Figure 7:**
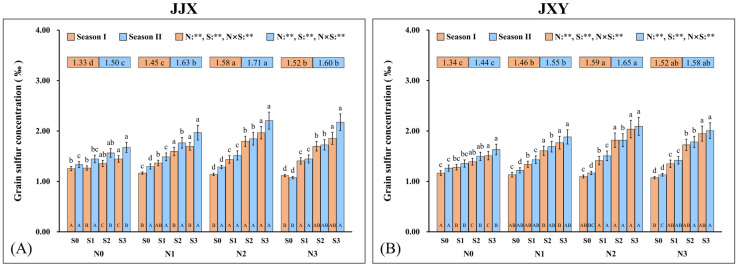
Grain S concentration of two rice varieties under the different N and S fertilization rates. (**A**) Grain S concentration of the JJX variety. (**B**) Grain S concentration of the JXY variety. The lowercase letters above the bars denote statistically significant differences between different S fertilization rates at the same N level, and the different uppercase letters in the bars denote statistically significant differences between different N fertilization rates at the same S level. The number within the top boxes of each set of bar charts denotes the average value of the grain S concentration at different N fertilization levels, and the lowercase letters after the number denote that the grain S concentration has statistically significant differences at different N fertilization levels. Variance analysis used two-way ANOVA, and ** denotes significance at *p* < 0.01.

Grain S concentrations are highly correlated with grain N accumulation (GPC). The experimental results of two consecutive planting seasons for the two varieties indicated that the fertilization of N and S exerts a significant impact on grain S concentration, with a significant interaction between the two elements (Figure 7). The grain S concentration of two planting seasons showed an initial increase (N2) and then a decrease (N3) with an increase in N fertilization rate, and the value of N3 in the JJX variety (1.52‰ and 1.60‰) was significantly lower than that of N2 (1.58‰ and 1.71‰), while there was no significant difference between the values of N3 (1.52‰ and 1.58‰) and N2 (1.59‰ and 1.65‰) in the JXY variety. Across all N application levels, grain S concentrations exhibited a consistent increase with rising S application rates. Compared to the S0 level, the JJX cultivar under the S3 level demonstrated increases in grain S concentration of 15.1% and 25.7% (N0); 45.5% and 51.8% (N1); 72.4% and 71.6% (N2); and 66.2% and 102.4% (N3) over two planting seasons, and the JXY cultivar under the S3 level showed increases of 29.6% and 29.2% (N0); 56.1% and 54.5% (N1); 85.8% and 79.2% (N2); and 81.6% and 77.4% (N3) across two planting seasons.

### 2.7. Grain Cysteine Concentration

Cysteine, a S-rich amino acid in rice grains, serves as the biochemical precursor for disulfide bond formation in proteins and exhibits a close correlation with GPC. Consequently, we conducted a determination and analysis of cysteine concentration in the grains. The experimental results indicated that N and S fertilization exerted a significant effect on grain cysteine concentration, with a notable interaction observed between these two elements. The grain cysteine concentration trends in both cultivars exhibit a highly concordant pattern, indicating that the regulatory effects of N and S on grain cysteine concentration are genotype-independent and universally applicable. Specifically, the grain cysteine concentration of both cultivars exhibited a consistent increase corresponding to the N fertilization rates. Compared with the non-N fertilization treatment (N0), the grain cysteine concentration of the JJX cultivar under N1, N2, and N3 levels increased by 17.1%, 28.7%, and 42.6%, respectively; likewise, the JXY cultivar exhibited increases of 17.4%, 39.1%, and 52.2%, respectively. Across all N application levels, the grain cysteine concentration of the two varieties exhibited a continuous increase with rising S fertilization rates, and both reached the maximum values at the S3 level. Under various N application levels, compared to the S0 level, the JJX cultivar under the S3 level demonstrated increases in grain cysteine concentrations of 34.9% (N0), 43.0% (N1), 64.0% (N2), and 75.4% (N3), and those of the JXY variety increased by 32.3%, 48.6%, 84.8%, and 81.5%, respectively.

### 2.8. Correlation Between Grain Protein Concentration and Cysteine Concentration

Cysteine plays a critical regulatory role in protein synthesis and stability maintenance. To further investigate the relationship between GPC and grain cysteine concentration, a correlation analysis was conducted in this study. The results showed a significant positive correlation between GPC and grain cysteine concentration, with highly consistent performance observed across both cultivars. The coefficient of determination (R^2^) for the JJX variety is 0.5215, whereas the JXY variety exhibits a higher coefficient of 0.6992. The direct manifestation of the positive correlation relationship observed in both cultivars demonstrates a high degree of consistency, indicating that the regulatory role of cysteine on GPC is genotype-independent and holds universal applicability. Based on the principles of protein biology and the relevant analysis, it can be concluded that the increase in grain cysteine concentration is the auxiliary or inducing reason for the increase in GPC.

## 3. Discussion

In contemporary agricultural production, nutrient management serves as a critical strategy to enhance the GY of crops [51]. In this study, the coordinated fertilization of N and S significantly increased the GY of rice (Figure 1). There is a quadratic curve between the GY and N fertilization rates, and high yields can be obtained at an optimal application amount. In this study, GY increased with an increase in N application from 0 kg ha^−1^ (N0) to 180 kg ha^−1^ (N2), while the N fertilization rate increased from 180 kg ha^−1^ (N2) to 240 kg ha^−1^ (N3). A significant decrease in GY was observed in both varieties (Figure 1). In a previous study, GY increased with the N fertilization from 0 to 195 kg ha^−1^, while the GY decreased with the N fertilization from 195 to 225 kg ha^−1^ [16], which is close to our research results. Furthermore, similar results were also observed in previous studies [52,53], indicating that excessive N fertilization has an inhibitory effect on GY. Under appropriate N application rates, the GY increased with the S fertilization rate from 0 kg ha^−1^ (S0) to 60 kg ha^−1^ (S3), but the GY in 60 kg ha^−1^ (S3) was not significantly higher than 45 kg ha^−1^ (S2), indicating that excessive S application no longer increased the GY in our study, which is consistent with other similar research [45]. Collectively, both supra-optimal and sub-optimal N and S application rates significantly reduced the GY of rice. Accordingly, the N2S2 combination is the optimal N and S nutrient management regimen for enhancing GY in rice.

The key to improving GY of rice lies in the effective coordination and optimization of yield components, such as the effective panicles, grain number per panicle, 1000-grain weight, and grain filling percentage. N and S fertilizers significantly influence the formation of yield components [45,54]. In this study, the effective panicles and grain numbers increased with rising N application rates, whereas the 1000-grain weight and grain filling percentage decreased (Table 1), which aligns with previously reported findings [55,56]. Excessive N application leads to a decline in grain weight due to delayed grain filling, which is a crucial factor in the reduction in GY of rice. Under appropriate N application rates, further S application coordinatively enhanced the effective panicles, grain numbers per panicle, 1000-grain weight, and grain filling percentage, which aligns with previous research findings [45].

Photosynthesis plays a critical role in the yield formation of crops, contributing approximately 70% of the biomass required for the GY of crops [57]. It has been demonstrated that improving the leaf photosynthetic capacity is pivotal for the accumulation of dry matter and GY in rice [58]. Improvements in the GY of rice are closely associated with variations in photosynthetic characteristics. In this study, the leaf net photosynthetic rate exhibits a continuous increase in response to escalating N application levels at each key growth stage of rice development (Figure 2). Previous research has demonstrated that N management plays a vital role in delaying leaf senescence and prolonging the duration of photosynthesis, which is positively correlated with an extended grain-filling period and increased yield [59,60]. Meanwhile, existing research has demonstrated that S application plays an important role in enhancing the photosynthesis of rice leaves [61]. In this study, under the premise of N application, combined S fertilization had a continuous promoting effect on the leaf photosynthesis of rice (Figure 2). It is particularly noteworthy that the synergistic fertilization of N and S significantly enhanced the leaf photosynthetic rate of rice from the grain filling stage to the maturation stage in our study. It has been established that enhancing photosynthesis during the grain filling stage increases GY by delaying leaf senescence and extending the duration of photosynthetic [47,62].

The dry matter production in plants constitutes a critically significant factor determining the GY formation of crops [63]. The accumulation and translocation of photosynthetic assimilates in various plant organs underpin dry matter production [64]. Previous research has demonstrated that the fertilization of N and S enhances GY by promoting dry matter accumulation [57,65]. In this study, the response of dry matter accumulation in plants to N and S application was highly similar to that of leaf photosynthetic rates (Figure 2 and Figure 3). The dry matter accumulation in plants exhibited a continuous increase with rising N application rates, and S application can further increase the dry matter accumulation of plants under N application. Typically, higher GY relies on substantial biomass production, as it is determined by the redistribution of dry matter accumulated in pre-anthesis vegetative organs, as well as the allocation of post-anthesis photosynthate [16]. However, optimal photosynthesis, as well as dry matter accumulation and distribution, necessitates adequate nutrient concentrations in the leaf tissue for sustained performance. In this study, in the absence of N application (N0), dry matter accumulation and photosynthesis significantly decreased with increasing S application rates, which can be attributed to the exacerbated nutrient imbalance induced by excessive sulfur supply. Under conditions without N application (N0), both the leaf N concentration (Figure 4) and GPC (grain N concentration) in rice (Figure 6) decreased progressively with increasing S application rates, indicating that S application inhibits N uptake in plants when N deficiency, thereby exacerbating nutritional imbalance. This phenomenon indicates that an appropriate ratio of N to S input is beneficial for nutrient balance and synergistic enhancement. The synergistic application of N and S enhanced leaf N and S concentrations at all key growth stages, particularly during the grain-filling stage (Figure 4 and Figure 5). The photosynthetic rate remains elevated during the post-anthesis phase, particularly in the middle to late stages of grain filling, promoting dry matter accumulation and grain development and ultimately enhancing the GY of crops. Optimal N and S application amounts would be beneficial to increasing dry matter accumulation and improving plant population quality, which could increase the grain weight, grain filling percentage, and GY of rice. 

**Figure 8:**
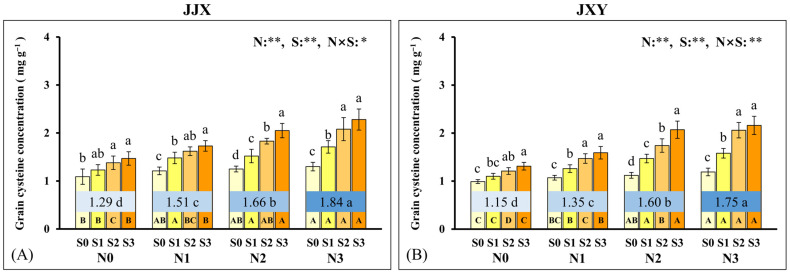
Grain cysteine concentration of two rice varieties under the different N and S fertilization rates. (**A**) Grain cysteine concentration of the JJX variety. (**B**) Grain cysteine concentration of the JXY variety. The lowercase letters above the bars denote statistically significant differences between different S fertilization rates at the same N level, and the different uppercase letters in the bars denote statistically significant differences between different N fertilization rates at the same S level. The number within the top boxes of each set of bar charts denotes the average value of the grain cysteine concentration at different N fertilization levels, and the lowercase letters after the number denote that the grain cysteine concentration has statistically significant differences at different N fertilization levels. Variance analysis used two-way ANOVA, ** denotes significance at *p* < 0.01, and * denotes significance at *p* < 0.05.

**Figure 9:**
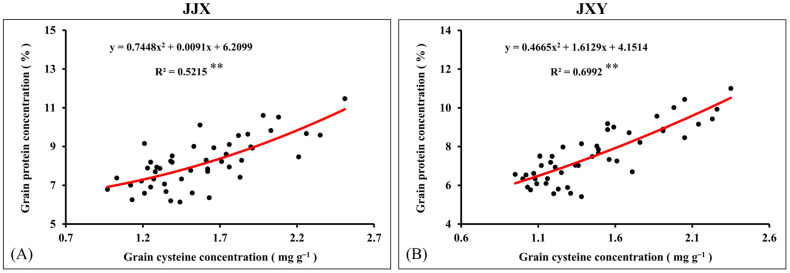
The correlation analysis between GPC and Cys concentration in rice grains. (**A**) JJX variety; (**B**) JXY variety. The red curve denotes the correlation between the GPC and Cys concentration in rice, and the black dots represent the GPC values at the corresponding Cys concentration in rice. ** indicates significance at *p* < 0.01.

As a crucial dietary source for humans, the GPC represents a core trait determining the nutritional quality of rice [6]. N and S are essential nutrient elements for protein synthesis, which are accumulated in proteins in the form of amino acids [66]. In this study, synergistic fertilization of N and S significantly enhanced both GPC and cysteine concentration in rice (Figure 7 and Figure 8). As a special amino acid containing both N and S, cysteine serves as the fundamental constituent for disulfide bond formation in proteins [67]. Disulfide bonds are covalent linkages formed through the dehydrogenation of sulfhydryl groups from two cysteine molecules, which are crucial for the correct folding of proteins. Once formed, the disulfide bonds can protect proteins by stabilizing their structural conformation and shielding sulfhydryl groups from excessive oxidation. In this study, the cysteine concentration in grains increased with an increase in N fertilization rates, and it further increased upon S co-supplementation, which was highly consistent with the change trend in GPC in rice. Given the constraints of complex protein structures, the elevated cysteine concentration provides additional potential sites for disulfide bond formation, which facilitates protein assembly and stability enhancement [68]. The positive correlation between GPC and cysteine concentration suggests an intrinsic biological relationship between them (Figure 9). Therefore, the elevated cysteine concentration constitutes the auxiliary or inducing reason contributing to the increased GPC in rice, which has been corroborated by our prior research on cereals such as maize [69,70]. It is worth emphasizing that the promotion of N assimilation and translocation by S application is the fundamental reason for the increase in GPC, which has been confirmed in crops such as rice and wheat [71,72]. In this study, the combined fertilization of N and S significantly promoted the absorption and assimilation of N by rice plants, which could be confirmed by the leaf N concentration (Figure 4). During the growth of rice plants, the leaves assimilate a substantial amount of N, reaching the highest levels at the flowering stage. Subsequently, the leaf N concentration decreases significantly from grain filling to the maturity stage, indicating the enormous translocation of N from leaves to grains.

## 4. Materials and Methods

### 4.1. Experimental Region Description

**Figure 10:**
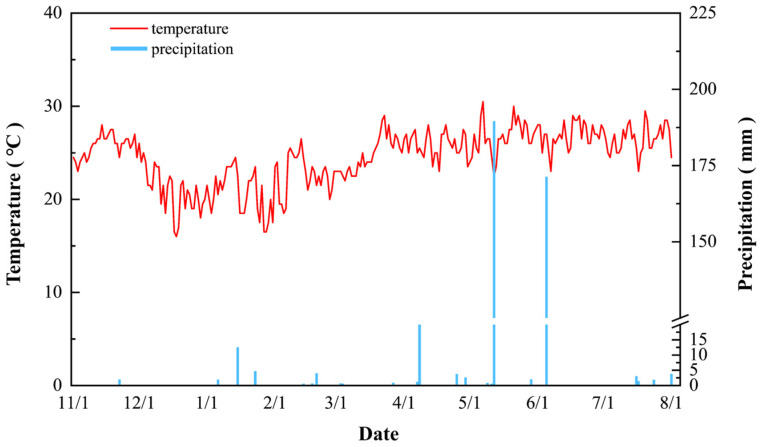
Temperature and precipitation at the experimental site during rice growing seasons from 2022 to 2023.

Consecutive two-planting-season field experiments were conducted from 2022 to 2023 at the Batou experimental base (N18°20′, E109°09′) in Hainan Province, China. The region features a tropical maritime monsoon climate, with an average annual temperature of 25.7 °C and an average annual precipitation of approximately 1350 mm. Detailed daily records of air temperature and precipitation during the rice growing seasons can be found in Figure 10.

The initial physical and chemical properties of the soil were determined according to the conventional method (Text S1) [73]. The initial physicochemical properties of topsoil (0–20 cm) from the experimental field are listed in Table 2. According to the textural classification, the soil type is categorized as sandy soil [74], with alkaline hydrolyzable N and available S contents of soil all residing at relatively low levels [70].

### 4.2. Experimental Design

The field experiment was a two-factor interaction design with different N and S application levels. Sixteen treatments of a combination of N and S and three repetitions for each treatment were undertaken. Four rates of N, i.e., no N (N0), low N (N1), moderate N (N2), and high N (N3), were applied. The amount of N application was 0, 120, 180, and 240 kg ha^−1^. Four rates of S, i.e., no S (S0), low S (S1), moderate S (S2), and high S (S3), were applied. The amount of S application was 0, 30, 45, and 60 kg ha^−1^. All treatments were applied with identical fertilizer rates of 70 kg ha^−1^ P_2_O_5_ and 90 kg ha^−1^ K_2_O. The N application ratio was 4:2:4 for the base, tillering, and panicle fertilizers. The potassium (K) application ratio was 6:4 for the base and tillering fertilizers. Phosphate (P) and S fertilizers are applied as base fertilizers in a single application prior to rice transplanting. The N, P, K, and S fertilizers used in the experiment were urea (N: 46%; Yuntian Chemical Co., Ltd., Kunming, China), monopotassium phosphate (P_2_O_5_: 52%; K_2_O: 34%; Chuanjinnuo Chemical Co., Ltd., Kunming, China, the K introduced was included in the total K application), potassium chloride (K_2_O: 60%; Senhai Chemical Co., Ltd., Geermu, China), and calcium sulfate dihydrate (S: 18%; Donghao Chemical Co., Ltd., Jining, China), respectively.

Two widely cultivated rice varieties in South China, Jiujiuxiang (JJX) and Jiuxiangyou (JXY), were used in this field experiment. The whole growth periods of JJX and JXY were 117–118 d and 114–115 d. The rice seeds were sown on 20 October 2022 and 20 March 2023 and then artificially transplanted on 15 November 2022 and 15 April 2023. Seedlings of homogenous size were manually transplanted with two plants per hill, and the planting density was 20 hill m^−2^. The experiments were conducted using a randomized complete block design, with each experimental plot measuring 30 m^2^ (5 × 6 m), and each treatment was repeated three times. The pest control measures and field management were carried out according to the requirements of local high-yield cultivation.

### 4.3. Determination of Yield and Its Components

At rice maturity, the average number of effective panicles was calculated by quantifying the effective panicles in 10 consecutive hills in each plot. Thereafter, six hills were selected from each plot to estimate the yield components [26]. In the central area of each plot, a 3 m^2^ rice section was demarcated for manual harvesting and threshing, and the GY of rice was adjusted according to 14.0% of the water content.

### 4.4. Determination of Photosynthesis

The net photosynthetic rate of rice leaves was measured by the portable photosynthesis system (Li-Cor, Inc., Lincoln, NE, USA) at the tillering, jointing, flowering, grain filling, and maturity stages. The measurements were performed during the morning of a sunny day (9:00–11:00). The leaf chamber’s headlight source was set to a light intensity of 1600 mmol m^−2^ s^−1^ photosynthetic photon flux density.

### 4.5. Determination of Plant Dry Matter

Six hills were randomly sampled from each plot at the tillering, jointing, flowering, grain filling, and maturity stages. Plant samples collected at the maturity stage were separated into stems (including leaves) and panicles. All plant samples were dried at 70 °C to a constant weight and then weighed.

### 4.6. Determination of N and S Concentrations in Leaves and Grains

According to the conventional method (Text S2) [47,73], all leaf and grain samples were oven-dried and ground into powder for the determination of nitrogen and sulfur concentrations. The samples of leaf and grain were digested with acid (H_2_SO_4_-H_2_O_2_), cooled to room temperature, and equilibrated with deionized water. Then, the concentration of N was measured by a Kjeldahl instrument (KETUO, Beijing, China). GPC was the N concentration multiplied by 5.95 [75]. After the samples of leaf and grain were digested with acid (HNO_3_-HClO_4_), the concentration of S was measured using an inductively coupled plasma instrument (SHIMADZU, Kyoto, Japan).

### 4.7. Determination of Cysteine Concentrations in Grains

According to our previous report, the cysteine concentration determination was performed using a high-performance liquid chromatography instrument (Agilent, Santa Clara, CA, USA) [70].

### 4.8. Data Analysis

All data across the N and S treatments were analyzed by two-way ANOVA using the Statistical Analysis System 9.2 (SAS Institute INC, Cary, NC, USA). Specifically, for the yield component data across the planting seasons, N and S treatments were analyzed for analysis of variance (ANOVA) using a three-way analysis program via SAS. The least significant difference (LSD) test was used to compare significant differences based on *p* values of < 0.05.

## 5. Conclusions

N and S exert significant effects on the GY and GPC of rice, with notable interactive effects between these two nutrient elements. The synergistic fertilization of N and S enhanced the GY by improving rice plant photosynthesis and dry matter accumulation while increasing GPC through elevated cysteine concentration in grains. Overall, for both the JJX and JXY cultivars, the fertilization of 180 kg ha^−1^ of N combined with 45 kg ha^−1^ of S (i.e., the N2S2 treatment) synergistically enhances GY and GPC in rice. Therefore, regulating N and S nutrient management measures provides a feasible strategy to simultaneously boost crop yield and nutritional quality, which is crucial for achieving sustainable food security without trade-offs. The synergistic application of N and S synergistically enhances both rice yield and nutritional quality by regulating plant growth dynamics, which meet the requirements for healthy and sustainable development in rice production systems and provide novel theoretical foundations alongside nutrient management strategies for achieving high-yield and premium-quality rice cultivation.

## Figures and Tables

**Table 1:** Grain yield components of two rice varieties under different N and S application rates.

Planting Season	Treatment		Variety							
			JJX				JXY			
			Effective Panicle	Grain Number	1000-Grain Weight	Grain Filling Percentage	Effective Panicle	Grain Number	1000-Grain Weight	Grain Filling Percentage
			(m^−2^)	(Panicle^−1^)	(g)	(%)	(m^−2^)	(Panicle^−1^)	(g)	(%)
Season I	N0	S0	161 a C	83 a C	28.58 a A	89.25 a A	154 a C	81 b D	27.69 a A	87.53 a A
		S1	164 a C	88 a C	27.54 ab A	89.04 a A	157 a C	83 ab D	26.81 ab A	88.49 a A
		S2	171 a C	89 a C	26.82 ab A	89.47 a A	163 a C	86 a C	26.01 ab AB	89.45 a A
		S3	171 a C	87 a C	26.17 b A	89.43 a A	166 a D	84 ab C	25.27 b A	90.59 a A
	N1	S0	209 b B	115 b B	23.76 b B	83.66 b B	183 b B	106 c C	23.58 b B	83.89 b B
		S1	219 ab B	121 ab B	25.22 ab B	86.33 ab AB	195 ab B	111 bc C	24.97 ab AB	85.58 ab AB
		S2	233 a B	128 a B	26.40 a A	88.05 a AB	212 a B	117 ab B	26.43 a A	88.37 a A
		S3	216 ab B	131 a B	26.18 a A	89.40 a A	202 a C	121 a B	26.21 a A	89.22 a A
	N2	S0	232 b A	127 b AB	22.18 b BC	79.80 b C	222 b A	116 b B	21.55 c C	78.15 b C
		S1	244 ab A	135 ab A	23.74 b BC	84.48 ab AB	236 ab A	125 ab B	23.06 bc BC	82.31 ab B
		S2	257 a A	143 a A	25.76 a A	87.33 a AB	250 a A	132 a A	24.79 ab AB	86.72 a A
		S3	258 a A	141 ab AB	25.87 a A	88.12 a A	254 a A	130 a AB	25.45 a A	87.98 a A
	N3	S0	225 a A	137 a A	20.21 c C	79.35 b C	210 a A	131 a A	19.61 c D	75.55 b C
		S1	228 a AB	141 a A	22.28 b C	82.52 ab B	218 a A	136 a A	21.61 b C	77.78 ab C
		S2	230 a B	144 a A	24.99 a A	83.90 a B	226 a B	140 a A	23.89 a B	80.15 ab B
		S3	230 a B	144 a A	24.70 a A	86.25 a A	229 a B	142 a A	24.77 a A	81.51 a B
Season II	N0	S0	144 a C	80 ab C	29.23 a A	91.26 a A	136 b C	77 b D	27.63 a A	86.06 a A
		S1	148 a C	86 a C	27.06 ab A	88.85 a A	143 ab C	82 ab C	26.44 ab A	87.49 a A
		S2	154 a C	83 ab C	26.21 b A	89.74 a A	154 a C	86 a C	25.31 ab AB	88.61 a A
		S3	148 a C	79 b C	25.52 b A	92.05 a A	149 ab C	83 ab C	24.42 b A	89.96 a A
	N1	S0	178 b B	103 b B	23.17 b B	84.41 c B	158 c B	100 b C	23.25 b B	81.09 c B
		S1	194 ab B	115 ab B	25.81 a A	86.07 bc A	180 b B	107 ab B	25.58 ab A	83.92 bc AB
		S2	203 a B	122 a B	26.34 a A	89.95 ab A	202 a B	110 ab B	26.64 a A	86.47 ab A
		S3	204 a B	114 ab B	24.35 ab A	91.68 a AB	186 ab B	113 a B	25.13 ab A	88.17 a A
	N2	S0	209 c A	124 a A	21.66 b BC	80.98 b B	208 b A	112 b B	20.94 c C	77.32 b BC
		S1	220 bc A	125 a AB	22.67 b B	86.51 ab A	221 ab A	120 ab A	22.85 bc B	81.06 ab BC
		S2	231 ab A	135 a A	24.78 a A	90.31 a A	243 a A	129 a A	25.35 ab AB	84.41 a AB
		S3	233 a A	137 a A	25.17 a A	90.15 a AB	247 a A	132 a A	26.15 a A	85.64 a AB
	N3	S0	196 a A	127 a A	20.87 c C	80.87 b B	192 b A	123 b A	18.31 c D	74.50 b C
		S1	205 a AB	134 a A	22.14 bc B	84.73 ab A	203 ab A	132 ab A	21.45 b B	76.94 ab C
		S2	211 a B	139 a A	24.01 ab A	86.52 ab A	218 ab AB	142 a A	23.91 a B	80.35 a B
		S3	214 a B	142 a A	24.34 a A	87.32 a B	221 a A	144 a A	24.61 a A	81.58 a B
ANOVA										
Season			**	**	ns	*	**	*	ns	*
N			**	**	**	**	**	**	**	**
S			**	**	**	**	**	**	**	**
Season × N			ns	ns	ns	ns	ns	ns	ns	ns
Season × S			ns	ns	ns	ns	ns	ns	ns	ns
N × S			ns	ns	**	*	ns	ns	**	ns
Season × N × S			ns	ns	ns	ns	ns	ns	ns	ns

Different lowercase letters after the data denote statistically significant differences between different S application rates at the same N level (*p* < 0.05), and different capital letters after the data denote statistically significant differences between different N application rates at the same S level (*p* < 0.05). ** denotes significance at *p* < 0.01, * denotes significance at *p* < 0.05, and ns denotes not significant (*p* > 0.05).

**Table 2:** The physicochemical properties of 0–20 cm topsoil at the experimental site.

Experimental Site	Soil Type	Soil pH	Organic Matter	Alkaline-N	Olsen-P	NH_4_OAc-K	Ca(H_2_PO_4_)_2_-S
			(g kg^−1^)	(mg kg^−1^)	(mg kg^−1^)	(mg kg^−1^)	(mg kg^−1^)
Batou Base	Sandy soil	6.13	9.4	84.93	21.85	167.01	10.58

## Data Availability

Data are contained within this article. Further inquiries can be directed to the corresponding authors.

## Supplementary Materials

The following supporting information can be downloaded at https://www.mdpi.com/article/10.3390/plants15071058/s1. Text S1: The specific analytical procedures for determining soil initial physicochemical properties; Text S2: The specific analytical procedures for determining N and S concentration in leaves and grains.
