# Supplementary material for: Coordinated Application of Nitrogen and Sulfur Synergistically Enhances Grain Yield and Grain Protein Concentration of Rice by Regulating Plant Growth
Source: Plants (Basel). 2026 Mar 30;15(7):1058. doi: 10.3390/plants15071058 (PMC13074531; doi:10.3390/plants15071058)
Supplement: Supplementary file 1 [file plants-15-01058-s001.zip › plants-4213431-supplementary.pdf]

plants

Supplementary materials

Coordinated application of nitrogen and sulfur synergistically enhances grain yield and grain protein concentration of rice by regulating plant growth

Honglin Wang [1,†], Jianan Fu [1,†], Huadong Gong [1], Linyan Kuang [1], Yuzhe Song [1], Zhaoyue Ma [1], Liqiang He [1], Bohan Xu [2], Shuai Cui [2], Shuoran Liu [1,2,*], Zhongqing Zhang [2,*] and Qiang Gao [2,*]

[1] School of Tropical Agriculture and Forestry, Hainan University, Haikou 570228, China
[2] College of Resource and Environmental Sciences, Jilin Agricultural University, Changchun, 130118, China
* Correspondence: shuoranliu@hainanu.edu.cn; zhangzhongqing@jlau.edu.cn; gaoqiang@jlau.edu.cn
† These authors contributed equally to this work.

Text S1: The specific analytical procedures for determining soil initial physicochemical properties [73]

(1) The **Soil pH** was determined by the soil–water suspension and a calibrated pH meter.

Procedure (1:5 Soil–Water Suspension):

Sample Preparation: Collect a representative soil sample from the field. Air-dry the sample at room temperature. Gently crush the aggregates and pass the soil through a 2 mm sieve. Remove any stones, roots, or debris. Mix the sieved soil thoroughly.

Preparation of the Suspension: Accurately weigh 10.0 g of the prepared air-dried soil into a clean 100 mL beaker. Using a measuring cylinder, add 50 mL of distilled or deionized water (free of CO_2 for most accurate results–achieved by boiling and cooling). Stir the mixture thoroughly with a glass rod for about 1–2 minutes to ensure all soil particles are wetted.

Calibration of pH Meter: Turn on the pH meter and allow it to warm up as per the manufacturer's instructions. Rinse the electrode with distilled water and gently blot it dry with soft tissue (do not rub). Calibrate the meter using at least two buffer solutions (e.g., pH 7.00 and pH 4.00). Start with pH 7.00. Immerse the electrode in the pH 7.00 buffer, stir gently, and allow the reading to stabilize. Set the meter to the correct value. Rinse the electrode with distilled water, blot dry, and repeat the process with the pH 4.00 buffer. The meter should now be accurately calibrated across the acidic range. For alkaline soils, a pH 10.00 buffer may be used as a third calibration point.

Measurement: Allow the soil suspension to stand for 30 minutes to allow for equilibration and to let most of the suspended clay particles settle. Stir the suspension briefly just before measurement. Rinse the electrode thoroughly with distilled water and blot dry. Immerse the electrode into the partially settled soil suspension, ensuring the glass bulb is covered by the solution but not touching the soil sediment at the bottom. Gently swirl the beaker or stir the suspension slowly and carefully to create a homogeneous mixture around the electrode. Wait for the reading on the pH meter to stabilize (this can take 30 seconds to a minute). Record the pH value to two decimal places.

(2) **The Content of Soil Organic Matter (SOM)** was determined by the Wet Oxidation Method (Walkley-Black). This is a classical chemical method that estimates SOM by oxidizing the organic carbon in the soil.

Procedure (Walkley-Black Method):

Sample Preparation: Air-dry the soil sample and pass it through a 0.15 mm sieve to remove roots and debris. Accurately weigh 0.1 to 0.5 g of the prepared soil (the amount depends on the expected organic matter content) into a hard glass tube or flask.

Oxidation: Add 10 mL of 0.4 mol L^{-1} potassium dichromate solution, followed by 20 mL of concentrated sulfuric acid. Swirl gently to mix. The mixture is often heated (e.g., in an oil bath at 180–185°C for 5 minutes or on a hot plate) to ensure complete oxidation.

Dilution and Titration: After cooling, transfer the mixture to a conical flask and dilute with distilled water to a volume of about 150 mL. Add a few drops of an indicator (o-phenanthroline). Titrate the solution with a standard ferrous sulfate solution until the color changes from blue-violet or orange-red to a green endpoint.

Blank Determination: Perform the entire procedure without a soil sample (using a small amount of silica or just the reagents) to determine the initial amount of dichromate.

Calculation: The content of SOC in the soil is calculated using the following formula:

$$SOC\ (g\ kg^{-1}) = [(V_{blank} - V_{sample}) \times M \times 10^{-3} \times 3 \times f] \times 100 / W$$

https://doi.org/10.3390/plants15071058

Where:

V_{blank} and V_{sample} are the titration volumes ($FeSO_4$) for blank and sample (mL),

M is molar concentration of the ferrous sulfate ($FeSO_4$) standard solution (mol L^{-1}),

10^{-3} is mL to L,

3 is the molar mass of carbon (1/4 C), expressed in grams (g). This factor is used because 1 mole of $K_2Cr_2O_7$ (based on its equivalent weight in this reaction) oxidizes 3 g of carbon,

f is a correction factor (often 1.1) to account for incomplete oxidation of organic carbon,

W is the weight of the soil sample (g).

SOM (g kg^{-1}) = SOC × 1.724

1.724 = The Van Bemmelen factor. This is the conventional factor used to convert organic carbon to organic matter, based on the assumption that soil organic matter contains 58% organic carbon (100/58 $\approx$ 1.724)

(3) **The Content of Soil available nitrogen (Alkaline-N)** was determined by the standard alkaline hydrolysis diffusion method.

Procedure (alkaline hydrolyzable nitrogen):

Sample Preparation: Air-dry a representative soil sample. Remove any visible debris (roots, stones). Grind the sample and pass it through a 2 mm sieve.

Weighing and Diffusion Dish Setup: Accurately weigh 2.00 g (±0.01 g) of the prepared air-dried soil sample. For soils with very high organic matter, a smaller sample (e.g., 1.00 g) may be used. Place the weighed soil into the outer ring of a clean, dry diffusion dish. Add approximately 0.2 g of finely ground ferrous sulfate powder to the soil in the outer chamber and mix thoroughly with a dry spatula. Pipette exactly 2.0 mL of the boric acid-indicator solution into the inner chamber of the diffusion dish (Take care not to spill any into the outer chamber). Apply the alkaline glue or silicone grease evenly to the flat rim of the diffusion dish (or the lid) to ensure an airtight seal. Using a syringe or pipette, quickly add 10.0 mL of the 1.8 mol L^{-1} NaOH solution to the soil in the outer chamber. Immediately cover the dish with its lid and press gently to create an airtight seal. The order of operations is critical to prevent ammonia loss. Swirl the dish gently to mix the soil and NaOH into a uniform paste.

Incubation: Place the sealed diffusion dishes in an incubator set at 40°C ± 1°C for a period of 24 hours.

Titration: After incubation, remove the dishes from the incubator and allow them to cool to room temperature. Titrate the boric acid solution in the inner chamber directly with the standard 0.01 mol L^{-1} HCl solution. Stir the solution gently during titration, either with a micro-stirrer or by carefully swirling the dish. The endpoint is reached when the color changes from the initial green (or blue-green) to a wine-red or violet-red. Record the volume of HCl used (V, in mL).

Blank Determination: Perform a blank determination alongside the samples, using the same procedure but without soil. All reagents are added in the same quantities. Record the blank titration volume (V_0, in mL).

Calculation: The content of alkali-hydrolyzable nitrogen in the soil is calculated using the following formula:

Alkali-hydrolyzable-N (mg kg^{-1}) = $[(V - V_0) \times C \times 14 \times 1000] / m$

Where:

https://doi.org/10.3390/plants15071058

V = Volume of standard HCl used for titrating the sample (mL).

V_0 = Volume of standard HCl used for titrating the blank (mL).

C = Exact concentration of the standard HCl solution (mol L^{-1}).

14 = Molar mass of nitrogen (g mol^{-1}).

m = Mass of the air-dried soil sample (g).

1000 = Conversion factor to express the result in mg kg^{-1}.

(4) **The Content of Soil Olsen-P** was determined by the sodium bicarbonate ($NaHCO_3$) extraction method.

Procedure (Olsen-P):

Soil Extraction: Accurately weigh 2.50 g of air-dried, ground (to pass a 2 mm sieve) soil into a 125 mL extraction bottle. Include a reagent blank (no soil) with each batch of samples. Add approximately 1 g of phosphorus-free activated charcoal to the bottle to adsorb organic matter and remove color from the extract. Then, add 50.0 mL of the 0.5 M $NaHCO_3$ extracting solution (pH 8.5) using a volumetric dispenser. Stopper the bottle securely and shake on a reciprocating shaker for exactly 30 minutes at a constant speed (approx. 180 cycles min^{-1}). Immediately after shaking, filter the suspension through a Whatman No. 42 filter paper into a clean, dry flask or beaker. The filtrate should be clear.

Color Development: Using a pipette, transfer an aliquot (e.g., 5.0 mL) of the clear soil extract, blank, and each working standard solution into separate 50 mL volumetric flasks or clean, dry test tubes. The aliquot volume should be chosen to ensure the final P concentration falls within the range of the standard curve. Slowly and carefully, add 5 mL of the 2.5 M H_2SO_4 solution to each flask/tube. This neutralizes the bicarbonate, which would otherwise interfere with the color reaction by raising the pH. It also acidifies the medium for the molybdate reaction. Gently swirl to allow the CO_2 bubbles to escape. Add 8.0 mL of the freshly prepared Mixed Reagent to each flask/tube. Make up the volume to the 50 mL mark (in flasks) with distilled water and mix thoroughly. If using test tubes, a fixed volume (e.g., to a 25 mL mark) can be used. Stopper and invert several times to mix. Allow the color to develop for at least 10 minutes but not more than 60 minutes. The blue color is stable for several hours.

Spectrophotometric Measurement: Set the spectrophotometer to a wavelength of 880 nm. If the instrument cannot read this high, 700 nm or 660 nm can be used, but 880 nm provides maximum sensitivity. Zero the spectrophotometer using the blank solution. Then, measure the absorbance of the working standard solutions. Plot the absorbance values (y-axis) against their corresponding P concentrations in mg P L^{-1} (x-axis) to create a standard curve. Calculate the linear regression equation. Measure the absorbance of all the unknown soil extract solutions. If any sample's absorbance is higher than the highest standard, the analysis must be repeated using a smaller aliquot of the soil extract or a dilution.

Calculations: The concentration of Olsen-P in the soil is calculated using the following formula:

Olsen-P (mg kg^{-1}) = ($C \times V_{ext} \times$ Dilution Factor) / W_{soil}

Where:

C = Concentration of P in the aliquot (from the standard curve) in mg L^{-1}.

V_{ext} = Volume of extracting solution used (L) = 0.050 L.

W_{soil} = Weight of air-dried soil (kg) = 0.0025 kg (2.50 g).

https://doi.org/10.3390/plants15071058

Dilution Factor = (Total volume of colored solution / Aliquot volume taken) × (any further dilution factor).

(5) **The Content of Soil Available Potassium (NH_4OAc-K)** was determined by the Ammonium Acetate (NH_4OAc) Extraction method using a Flame Photometer.

Procedure (NH_4OAc-K):

Preparation of Soil Sample: Air-dry the collected soil sample at room temperature. Grind the dried soil with a wooden mallet or porcelain mortar and pestle. Pass the ground soil through a 2 mm stainless steel sieve. Mix the sieved soil thoroughly and store it in a labeled, clean container.

Extraction (Shaking): Accurately weigh 5.0 g of the prepared 2 mm air-dried soil sample into a clean 100 mL conical flask. Add 25 mL of the 1N Ammonium Acetate (pH 7.0) solution to the flask using a pipette or volumetric dispenser. Stopper the flask securely and place it on a mechanical shaker. Shake for 30 minutes at a constant speed (approx. 180-200 rpm). After shaking, immediately filter the suspension through Whatman No. 1 filter paper into a clean, dry conical flask or beaker. The filtrate should be clear.

Preparation of Blank: Prepare a "blank" solution by taking 25 mL of the pure 1N Ammonium Acetate extracting solution (without soil) through the same filtration process. This blank accounts for any trace potassium in the reagents and will be used to zero the flame photometer.

Measurement using Flame Photometer: Turn on the flame photometer and allow it to stabilize. Set the gas and air pressure according to the manufacturer's manual. Insert the potassium filter. Aspirate (suck up) the blank solution (0 ppm standard) into the instrument and adjust the reading to zero. Aspirate the highest standard (100 ppm) and adjust the sensitivity control to get a full-scale deflection (e.g., reading of 100). Aspirate the intermediate standards (20, 40, 60, 80 ppm) and record their readings. Aspirate the blank again to ensure the reading has not drifted.

Sample Measurement: Aspirate the soil sample filtrates one by one. Record the stable reading (emission intensity) for each sample. If a sample reading is higher than your highest standard (100 ppm), it needs to be diluted. Take an aliquot (e.g., 5 mL) of the filtrate, add a known volume (e.g., 5 mL) of the 1N Ammonium Acetate solution, mix, and re-analyze. Remember to multiply by the dilution factor in the final calculation.

Calculation: Prepare a graph by plotting the flame photometer readings (on the Y-axis) against the standard potassium concentrations in ppm (on the X-axis). This should yield a straight-line (linear) graph. Determine Sample Concentration: Using the reading obtained for your unknown sample, find the corresponding concentration of potassium (C) in ppm (mg L^{-1}) from the standard curve.

The concentration of Available K in the soil is calculated using the following formula:

Available K (mg kg^{-1}) = (C × V × Dilution Factor) / W × 1000

Where:

C = Concentration of K in the filtrate read from the curve (mg L^{-1}).

V = Total volume of extracting solution used (L). Here, 25 mL = 0.025 L.

Dilution Factor = Applied only if the sample was diluted (if not diluted, DF = 1).

W = Weight of the soil sample (kg). Here, 5 g = 0.005 kg.

1000 = Factor to convert mg to kg for the final unit.

https://doi.org/10.3390/plants15071058

(6) **The Content of Soil Available Sulfur [$Ca(H_2PO_4)_2$-S]** was determined by the Calcium Dihydrogen Phosphate [$Ca(H_2PO_4)_2$] Extraction method using an Inductively Coupled Plasma Emission Spectrometry (ICP).

Procedure [$Ca(H_2PO_4)_2$-S]:

Sample Preparation and Extraction: Air-dry the soil sample and gently grind it to pass through a 2 mm sieve. This ensures homogeneity and prepares the soil for consistent extraction. Accurately weigh 5.00±0.01 g sample of the prepared soil into a clean extraction bottle. Add 50 ml of 0.5 mol L^{-1} calcium dihydrogen phosphate ($Ca(H_2PO_4)_2$) solution to the extraction bottle. Securely cap the bottle and shake it for 30 minutes in a mechanical shaker at a controlled temperature of 25°C to ensure equilibrium and complete extraction of the available sulfate. Immediately after shaking, filter the suspension through a Whatman 40 filter paper, discarding the first few milliliters of filtrate to avoid any contamination from the filter paper. The filtrate is collected in a clean container.

Instrumental Analysis: The ICP instrument should be allowed to warm up and stabilize, typically for at least 2 hours, to minimize signal drift. The plasma is ignited, and parameters like nebulizer gas flow, auxiliary gas flow, and RF power are optimized for the analysis of sulfur in the prepared matrix.

Wavelength Selection: A commonly used and sensitive line for sulfur is 182.034 nm (or 182.037 nm).

Calibration: Prepare a series of calibration standards using the same background solution ($Ca(H_2PO_4)_2$) as the samples. The concentration range should encompass the expected sulfur concentrations in the samples (e.g., 0-40 mg L^{-1}). The instrument software plots the emission intensity versus concentration to generate a calibration curve, which must have a high correlation coefficient (e.g., $r > 0.999$).

Sample Analysis: The treated and diluted sample extracts are introduced into the ICP. The instrument measures the emission intensity at the selected sulfur wavelength. The concentration of sulfur in the analyzed solution is automatically calculated from the calibration curve.

Calculations: The concentration of available sulfur in the soil is calculated using the following formula:

Available S (mg kg^{-1}) = $[(C - C_0) \times V \times f] / m$

Where:

C = Concentration of sulfur in the sample extract solution (mg L^{-1}), as measured by ICP.

C_0 = Concentration of sulfur in a method blank solution (mg L^{-1}).

V = Total volume of the extracting solution used (L). Here, 50.0 mL is 0.050 L.

f = Dilution factor, if the sample extract was diluted further after the initial treatment (if no dilution, f = 1).

m = Mass of the soil sample (kg). Here, 5.00 g is 0.005 kg.

https://doi.org/10.3390/plants15071058

Text S2: The specific analytical procedures for determining N and S concentration in leaves and grains [47, 73]

(1) **The Concentration of N in leaves and grains** was determined by the acid (H_2SO_4-H_2O_2) Digestion method using a Kjeldahl instrument.

Sample Preparation: Fresh plant material (leaves and grains) should be thoroughly dried, typically in an oven at 70°C until a constant weight is achieved. Grinding: The dried tissue must be ground to a fine powder to ensure a homogeneous sample. A common specification is to pass the material through a 0.42 mm sieve. This increases the surface area for efficient digestion.

Digestion: An accurately weighed sample, typically between 0.10 g and 0.20 g, is placed into a Kjeldahl digestion tube. Add 5 ml of concentrated sulfuric acid (H_2SO_4) and 2 ml of hydrogen peroxide(H_2O_2) to the digestion tube containing the sample. The tubes are placed in a digestion block or over heaters and heated. The temperature is carefully controlled, often in stages or maintained at a high temperature (360-380°C). The digestion continues until the mixture becomes clear, indicating that all organic matter has been broken down. This can take from 2 to 3 hours after clearing, depending on the protocol.

Cooling and Dilution: After digestion, the tubes are removed and allowed to cool. A precise volume of distilled water is added to dissolve the digestate and dilute the acid, bringing the total volume up to a known mark (100 mL). This is the digest solution used for analysis.

Distillation (Isolating Ammonia): An aliquot of the plant digest (25 mL) is transferred to the distillation apparatus. A strong base, usually 40-50% sodium hydroxide (NaOH), is carefully added (4 mL) to make the solution highly alkaline. This converts the ammonium ions (NH_4^+) in the digest into ammonia gas (NH_3). The Kjeldahl distillation unit passes steam through the alkaline mixture. The steam carries the volatile ammonia gas out of the distillation flask and through a condenser, where it is cooled and condensed. The condensed liquid, containing the ammonia, is distilled directly into a receiving flask. The receiving flask contains a known volume of a boric acid (H_3BO_3) solution with an indicator mix (bromocresol green and methyl red). The ammonia reacts with the boric acid to form ammonium borate, which causes the solution to change color (from purple to green), indicating that the ammonia is being trapped. Distillation continues until a sufficient volume of distillate (20-25 mL) has been collected. The ammonium borate in the receiving flask is then titrated with a standard acid, typically 0.1 N sulfuric acid (H_2SO_4). The acid reacts with the ammonium borate, and the endpoint of the titration is reached when the solution returns to its original acidic color (from green back to purple).

Calculation: The volume of acid used in the titration is directly proportional to the amount of nitrogen in the sample. The nitrogen concentration is calculated using the following formula:

Nitrogen concentration (%) = $[(V_{sample} - V_{blank}) \times N \times 14.01] / W$

Where:

V_{sample} = Volume of titrant (H_2SO_4) used for the sample titration (mL).

V_{blank} = Volume of titrant (H_2SO_4) used for the blank titration (mL).

N = Normality of the titrant (in equivalents per liter).

14.01 = Atomic mass of nitrogen (g eq^{-1}).

W = Weight of the sample (g).

(2) **The Concentration of S in leaves and grains** was determined by the acid (HNO_3-$HClO_4$) digestion method using an Inductively Coupled Plasma Emission Spectrometry.

Digestion: An accurately weighed sample, typically between 0.10 g and 0.15 g, is placed into a digestion tube. Add 5 ml of a mixture of concentrated nitric acid and perchloric acid (HNO_3-$HClO_4$, v/v, 4:1) to the digestion tube containing the sample. The digestion tubes are placed in a digestion block or over heaters and heated. The temperature is carefully controlled, often in stages or maintained at a high temperature (340°C). The digestion continues until the mixture becomes clear, indicating that all organic matter has been broken down. This can take from 1 to 2 hours after clearing, depending on the protocol.

Cooling and Dilution: After digestion, the digestion tubes are removed and allowed to cool. A precise volume of distilled water is added to dissolve the digestate and dilute the acid, bringing the total volume up to a known mark (100 mL). This is the digest solution used for analysis.

Instrumental Analysis: The ICP instrument should be allowed to warm up and stabilize, typically for at least 2 hours, to minimize signal drift. The plasma is ignited, and parameters like nebulizer gas flow, auxiliary gas flow, and RF power are optimized for the analysis of sulfur in the prepared matrix.

Wavelength Selection: A commonly used and sensitive line for sulfur is 182.034 nm.

Calibration: Prepare a series of calibration standards using the same background solution (HNO_3-$HClO_4$) as the samples. The concentration range should encompass the expected sulfur concentrations in the samples (0-50 mg L^{-1}). The instrument software plots the emission intensity versus concentration to generate a calibration curve, which must have a high correlation coefficient ($r > 0.999$).

Sample Analysis: The treated and diluted sample extracts are introduced into the ICP. The instrument measures the emission intensity at the selected sulfur wavelength. The concentration of sulfur in the analyzed solution is automatically calculated from the calibration curve.

Calculations: The sulfur concentration of leaves and grains are calculated using the following formula:

Sulfur concentration (mg/g) = $[(C - C_0) \times V \times f] / m$

Where:

C = Concentration of sulfur in the sample digest solution (mg L^{-1}), as measured by ICP.

C_0 = Concentration of sulfur in a method blank solution (mg L^{-1}).

V = Total volume of the digest solution used (L). Here, 100 mL is 0.1 L.

f = Dilution factor, if the sample extract was diluted further after the initial treatment (if no dilution, $f = 1$).

m = Mass of the sample (g). Here, 0.10 g to 0.15 g.

https://doi.org/10.3390/plants15071058
